# A Study of the Periods of Incubation of the Communicable Diseases*Read before the Association of Medical Officers of Health, Friday, May 20th, 1881.

**Published:** 1882-07

**Authors:** Benjamin Ward Richardson


### A STUDY OF THE PERIODS OF INCUBATION OF THE COMMUNICABLE DISEASES.* BY BENJAMIN WARD RICHARDSON, M.D., F.R.C.P., F.R.S.

The points I propose for discussion are the following:

1. The precise application of the term incubation, and to what diseases it should be applied?

2. The amount of absolute knowledge of the period of incubation of the more common communicable diseases?

3. The differences of opinion on this subject; do they depend on fault of observation, or on the circumstance that there are, in the same diseases, varying periods of incubation, the variations being under no, as yet, defined rule?

4. If it be seen that there are differences of periods in the same diseases, upon what may such differences depend?

5. The practical lessons which arise from the facts we at present possess, and the extent to which our knowledge may be rendered available in regard to direction in the prevention and treatment of disease?

The period in the course of a disease to which we should apply the term incubation, and the diseases to which we should strictly apply the term, are the two first points deserving consideration.

At the outset this may seem to be a subject easy enough to settle, but when the mind comes down to it, it is not at all easy. We simplify somewhat by saying that the term is to be limited to those instances in which, after a certain specific poisonous particle has been taken into a living body, certain indications of the action of that specific poisonous matter has been manifested. We say that the period between the introduction of the poisonous particle, and the appearance of manifestation of action, is the period of incubation, but we fail to be unanimous on the question —What is the accepted first sign of such manifestation? Is that first sign any sign, or one particular sign? Is it a local sign, or is it a general or systemic sign? If, again, it be a systemic sign, is it one systemic sign, or two, or more, or one of many?

Let me give a small illustration of what I mean. The older

* Read before the Association of Medical Officers of Health, Friday, May 20th, 1881.

pracitioners, when they inoculated small-pox, ruled that the orange-colored eruption around the needle-puncture came on from the fourth to the fifth day, followed by a distinct vesicular rising, with pain in the axilla, on the seventh day, and systemic pain—pain in the back and limbs, and head—with rigor and fever on the ninth or tenth day, and appearance of a general eruption on the eleventh day. What here was the period of incubation ? Was it from the time of the insertion to the occurence of pain in the axilla, or to the rigor with pain in the back and the limbs, or to the general eruption ? In vaccinia in these days, we meet with the question—Is the appearance of the local eruption at the point of puncture, and the formation of the vesicle, to be counted as the end of the period of incubation ; or is the after fever, or other systemic symptom, to be taken as the close of the period ? I have known a case of vaccinia in which there has been no indication of effect at all, except the local manifestation. In such a case am I to say that the local change is sufficient when it is vesicular, when it is pustular, when it is maturative ? Or am I, in such a case, to say there has been no period of incubation at all ; that the whole process is abortive, and that the vaccinated is therefore either insusceptible or unprotected ? I take this illustration because it is so very definite, because we know that the poisonous particle was inserted at a certain time, and can therefore be traced from that time into effect. When we come to the question of reception of the poison by a means less definite, we have more reason than ever for some decided rule. In scarlet fever what shall guide us ? Shall it be the actual fact of a change in temperature ? If so, shall it be preliminary depression of temperature ? Or shall it be the first elevation of temperature that follows depression ? Or, shall it be pain, or shall it be eruption ? Until we can agree on these points, there can never be accuracy in our records.

I have studied very closely for two years past the various sides which this question presents, and by exclusion I have, for the moment, come to an opinion I am about with submission to offer to you. I would exclude all mere local manifestations, because I see that they may be presented and may not indicate that the disease has been incubated, in the full sense of that term. I exclude pain, local or general, because that might be due to other causes than the action of the poisonous material, or if it were due to that it is too much of a subjective symptom to be reliable. I exclude eruption because it may not be present, although the disease may be incubated, or it may be looked over, or it may be transient and therefore not detected. I exclude mere rise of temperature because that alone might not be detected at its origin, but might have been long present before it was detected. But I accept as the safest of all the phenomena indicating that the period of incubation is declared, that sudden vibration between cold and heat, which we will call chill or rigor. This is at once a subjective and an objective phenomenon. The observer sees it in the pallor and coldness and shivering, and reactive fever of the affected. The subject of the attack is easily able to define its precise period of commencement and its course. Moreover, of all the phenomena it is the most certain. In my experience I never knew a case of a communicable disease that was not preceded by chill. In cholera, in which it might perhaps not be expected by those who have not seen the disease, according to my experience in the two epidemics I have witnessed, it was always present. "The chill came on,"at such a time, was nearly the invariable sentence which fell from the lips of the stricken who were able to describe their own sufferings. There is moreover, a physiological reason for accepting chill or rigor as the indication that the poisonous particle has taken effect. The phenomenon is the natural indication that the disunion of normal action between the nervous and the vascular systems has taken place. It is like to the convulsive tremor which occurs when at a precise point during loss of blood the break between blood supply and nervous supply is interrupted. It is still more like the tremor which is invariably produced when an alkaloidal poison, like nicotine, shows its diffusion through the body, and, as we would say, declares that the period of its introduction is finished. If we had an alkaloid like nicotine which would increase in the body and by its increase diffuse up to the pitch of physiological action, we should rule that the incubation was finished when the tremor declared it. Granted then, that a disease runs a definable course, I should take the first rigor or chill as the first sign of the disease, and should accept the intervening period between the introduction of the cause and the developement of the chill, as the true period of incubation.

In the next place we may study under the present head the diseases to which the term period of incubation may be properly applied. On this point there is nothing strictly definite amongst the members of the profession. We are probably pretty unanimous in assuming a stage of incubation to the following common affections, 25 in number:—

1. Plague.
2. Cholera.
3. Malignant pustule.
4. Scarlet fever.
5. Diphtheria and croup.
6. Influenza.
7. Pertussis.
8. Glanders.
9. Erysipelas.
10. Farcy.
11. Grease.
12. Dengue.
13. Gonorrhœa.
14. Relapsing fever.
15. Variola.
16. Vaccinia.
17. Varicella.
18. Measles.
19. Rotheln.
20. Typhus.
21. Typhoid.
22. Mumps.
23. Malarial fever.
24. Syphilis.
25. Hydrophobia.

But there are some others on which we should not be so unanimous, as for instance—

Catarrh.
Puerperal fever.
Pyæmia.
Hospital gangrene.
Sloughing phagedæna.
Phagedæna.
Remittent fever.
Intermittent fever.
Choleraic diarhœa.
Cerebro-spinal fever.
Carbuncle.

These last-named diseases all require to be more carefully observed in respect to period of incubation and first indication of defined symptoms before they can be proved to have a stage of incubation at all, and before, therefore, our knowledge can be said to be perfect in respect to them.

I would now venture to indicate what are the nearest approaches to the periods of incubation in the different diseases which present an undoubted stage of incubation. In preparing the chapters of a work on preventive medicine, I thought it worth the trouble to collate the views of all our best authorities on the subject, old and modern, and to bring them into a condensed form, adding thereto what I had myself observed from natural facts.

Without loss of time I will state the results in brief form, but I must not let the opportunity pass of expressing how deeply we are indebted to Dr. Squire for the insight he has shown in dividing the communicable diseases according to their periods of incubation. Following closely his admirable plan, but somewhat extending it, I divide the diseases attended with stages of incubation into five groups:—

*a.* *Shortest.*—Shortest Stage of Incubation—1 to 4 days.—Malignant cholera. Malignant pustule. Plague. Catarrh. Dissection wound diseases.

*b.* *Short*—Second Short Period—2 to 6 days.—Scarlet fever. Rosalia idiopathica. Diphtheria. Dengue. Erysipelas. Yellow fever. Pyæmia. Influenza. Pertussis. Glanders. Farcy. Grease. Croup. Puerperal fever.

*c.* *Medium.*—Medium Periods—5 to 8 days.—Relapsing fever. Gonorrhœa. Vaccinia. Inoculated variola.

*d.* *Long.*—Long Periods—10 to 15 days.—Small-pox. Varicella. Measles. Rötheln. Typhus. Typhoid. Mumps. Malarial fever.

*e.* *Longest.*—Longest Period—40 days or more.—Syphilis. Hydrophobia.

If we may accept these as the common expression of knowledge up to the present time we have still much to learn. We exclude several diseases altogether which probably have some stage of incubation, and we are obliged to grant exceptions in respect to those that admit of being more readily classed together.

[TO BE CONCLUDED IN AUGUST NUMBER.]

